# A. V. M. A.

**Published:** 1902-05

**Authors:** 


### A. V. M. A.

President Winchester has appointed the following members, Local Committee of Arrangements for the annual meeting to be held in Minneapolis: Drs. C. C. Lyford, Chairman; S. D. Brimhall, M. H. Reynolds, J. G. Annand, J. S. Butler, J. N. Gould, A. Youngberg, S. H. Ward and K. J. McKenzie.

The Local Committee has met and organized, forming several sub-committees, and is actively at work arranging to entertain the largest number of veterinarians ever gathered together on American soil. Members of the committee are visiting State Associations and endeavoring to enlist interest in and support of this meeting.

### NECROLOGY.

John R. Comstock, veterinary surgeon, Pittsburg, Pa., died at the West Penn Hospital, in March, at the age of fifty-six years. He is survived by a wife and daughter.

### MARRIAGES.

Dr. John Anderson, of Seward, Nebraska, surprised his many friends by taking unto himself a better-half in the person of Miss Myrtle Boyer, of his city. The veterinarians of Nebraska had considered the doctor a confirmed bachelor, and unanimously congratulate him upon joining the army of Benedicts. The doctor and his bride started on their wedding tour with the cannon booming, and the citizens shouted words of good cheer, and upon their return were promised an old-time charivari. The wedding trip included a week's sojourn in Kansas City.

Dr. William H. Lowe, of Paterson, N. J., undaunted by the great conflagration that caused the loss of his entire hospital, library, and contents, continued to direct the fight in the New Jersey State Legislature for a better law regulating veterinary practice in that Commonwealth.

